# The Inflammation-Mediated Bidirectional Relationship Between Cardiovascular Disease and Cancer

**DOI:** 10.3390/diseases14070237

**Published:** 2026-07-02

**Authors:** Shahzaib Chughtai, Shofikur Shuhag, Daksh Saksena, Manum Zaman, Muhammad Usman Ghani

**Affiliations:** 1 College of Medicine, Central Michigan University, 1632 Stone St, Saginaw, MI 48602, USA; 2 Henry Ford St. John, 22101 Moross Rd, Detroit, MI 48236, USA; shuha1sr@cmich.edu; 3 Johns Hopkins Medicine, 600 N. Wolfe Street, Baltimore, MD 21231, USA; dsaksen1@jh.edu; 4 Fatima Memorial Hospital College of Medicine and Dentistry, Shadman Rd, Ichhra Lahore 54000, Pakistan; zamanmanum24@gmail.com; 5 MyMichigan Medical Center, 800 S. Washington Avenue, Saginaw, MI 48601, USA; ghani1mu@cmich.edu

**Keywords:** atherosclerotic cardiovascular disease, cardiology, oncology, inflammation, oxidative stress, reverse cardio-oncology

## Abstract

Cancer and atherosclerotic cardiovascular disease (ASCVD) represent two of the leading causes of death worldwide. Increasingly, these two are being recognized as biologically related conditions rather than entirely segregated disease states. In addition to traditional risk factors such as aging, smoking, and obesity, chronic inflammation may be a key factor connecting the two illnesses. Endothelial dysfunction, oxidative stress, plaque progression, and thrombosis are all facilitated by inflammatory signaling in ASCVD. Similar pathways are known to contribute to cancer growth and invasion. Emerging epidemiologic data demonstrate increased cancer incidence among patients with cardiovascular disease, while cancer survivors and recipients of cardiotoxic therapies exhibit accelerated vascular disease. This narrative review aims to describe the bidirectional relationship between ASCVD and cancer. Targeting shared pathways using statins, colchicine, canakinumab, IL-6 inhibition, and lifestyle modification may provide dual benefits. Future biomarker-guided trials with integrated cardiovascular and oncologic endpoints are needed to clarify causality and optimize prevention and management.

## 1. Introduction

Cardiovascular disease (CVD) and cancer are the two leading causes of death globally. In 2023, CVDs were responsible for 19.2 million deaths and 437 million disability-adjusted life years (DALYs) [1], while cancer (excluding non-melanoma skin cancers) accounted for 10.4 million deaths and 271 million DALYs globally [2]. Multiple traditional risk factors are shared between atherosclerotic CVD (ASCVD) and cancer, including smoking, aging, obesity, hypertension, and diabetes [3]. Common cancer therapies such as chemotherapy have well-established cardiotoxic effects. By exploring the pathological mechanisms common to CVD and malignancy, we lay a potential groundwork to explore therapies that are beneficial for both conditions.

Large-scale epidemiological studies have identified a 13% increased risk of cancer for CVD patients compared with patients who do not have prior CVD, accounting for traditional risk factors [4]. Likewise, survivors of cancer at various sites demonstrated accelerated atherosclerosis and increased medium- to long-term risk for CVD [5]. The bidirectional relationship may be at the center of emerging “cardio-oncology” and “reverse cardio-oncology” research, which explore how cancer therapies affect cardiovascular health and how cardiovascular disease influences cancer development, respectively [6]. Reverse cardio-oncology examines how cardiovascular disease may promote cancer development [7].

Accumulating evidence has supported a fundamental biological connection mediated by, among other processes, chronic inflammation [8]. In the case of malignancy, inflammation may lead to tissue stress and damage, facilitating cancer invasion and metastasis. This tissue stress may also contribute to vascular narrowing and ASCVD [8]. Inflammatory stress of the vascular wall promotes atherosclerotic plaque initiation, progression, and destabilization, while also potentially supporting a microenvironment for malignant transformation and tumor growth [9,10]. Establishing a causal relationship between inflammation and both atherosclerosis and cancer has important implications for clinical management.

This narrative review aims to elaborate on the potential role of inflammation in the association between ASCVD and cancer, with an emphasis on precise molecular mechanisms and clinical implications. This may establish an organized framework of ASCVD-cancer pathologic processes for treatment modulation and future research.

## 2. Materials and Methods

A focused literature review was conducted following a protocol registered on Open Science Framework prior to manuscript completion [https://osf.io/h4qaw/overview?view_only=af92931390ff484bb866f23ff9a629e7] (accessed on 27 April 2026). PubMed and Scopus databases were searched using Boolean search strategies designed to capture the breadth of the ASCVD-cancer-inflammation literature. Supplemental searching included manual review of reference lists from key articles, guideline bibliographies, and forward citation searching of landmark studies.

Search Strategy 1

(“cardiovascular disease” OR CVD OR atherosclerosis OR “heart failure”) AND (cancer OR neoplasm<em>OR malignan</em>) AND (“shared risk factors” OR “common mechanisms” OR “cardio-oncology” OR bidirectional OR association) AND (inflammation OR cytokines OR “shared mechanisms” OR biomarkers OR “cardio-oncology”)

PubMed: 8837 results; Scopus: 7079 results.

Search Strategy 2

(inflammation OR “chronic inflammation” OR cytokines OR “IL-6” OR “CRP” OR “TNF-alpha” OR “TNF-α” OR secretome OR CHIP OR “clonal hematopoiesis” OR “oxidative stress”) AND (“cardiovascular disease” OR atherosclerosis OR “coronary artery disease” OR ASCVD) AND (cancer OR neoplasm<em> OR tumorr OR malignan</em>) AND (mechanism OR pathogenesis OR complications OR “adverse effects”)

PubMed: 12,610 results; Scopus: 22,919 results.

Search Strategy 3

(“cancer therapy” OR chemotherapy OR “antineoplastic agents” OR immunotherapy OR “targeted therapy” OR “immune checkpoint inhibitors”) AND (cardiotoxicity OR “cardiovascular toxicity” OR “cardiac dysfunction” OR “heart failure” OR ASCVD OR atherosclerosis OR “vascular injury”) AND (mechanism OR pathogenesis OR complications OR “adverse effects”) AND (“shared risk factors” OR “common mechanisms” OR “cardio-oncology” OR bidirectional OR association)

PubMed: 28,712 results; Scopus: 2651 results.

Inclusion criteria: Studies were included if they met one or more of the following: (1) human clinical studies (randomized controlled trials, cohort studies, case–control studies, registries); (2) meta-analyses or systematic reviews relevant to the ASCVD-cancer-inflammation relationship; (3) animal or mechanistic preclinical studies explaining shared pathways; or (4) guidelines or scientific statements from recognized medical societies. Topically, studies were required to address at least one of the following: ASCVD and cancer association, reverse cardio-oncology, cancer therapy–induced vascular disease, shared inflammatory biomarkers, or anti-inflammatory interventions relevant to both diseases. The search was limited to English-language publications from 2010 onward.

Exclusion criteria: Studies were excluded if they were: (1) published before 2010 unless considered a landmark historical study; (2) published in a non-English language; (3) limited solely to heart failure without relevance to ASCVD; (4) limited solely to malignancy without cardiovascular linkage; (5) editorials lacking substantive data; (6) case reports unless uniquely mechanistic; or (7) duplicate publications.

Screening process: Titles and abstracts were screened for relevance to the review questions. Articles meeting criteria were retrieved in full text. Given the narrative review design, formal risk-of-bias assessment was not performed; however, the level of evidence for each study was noted and considered during review.

## 3. Inflammation in Atherosclerotic Cardiovascular Disease

Atherosclerosis is precipitated by inflammatory disease of the arterial wall. Multiple different cell types, including endothelial cells, smooth muscle cells, macrophages, and T lymphocytes, act through interconnected signals leading to atherosclerosis [11]. Traditional risk factors trigger endothelial dysfunction, leading to oxidative modification and retention of lipoproteins within the vessel intima. A comprehensive synthesis by Libby P. et al. discussed how these modified lipoproteins activate innate immune responses and recruit monocytes, which differentiate into macrophages and engulf the oxidized lipids to become foam cells [12]. The authors emphasize that both innate and adaptive immune effector mechanisms contribute to the pathogenesis of atherosclerosis, and that these immune pathways link many traditional cardiovascular risk factors to altered arterial function.

The vascular wall is susceptible to oxidative damage through reactive oxygen species (ROS) producing systems. A review by Förstermann U. et al. synthesized components in these systems to include NADPH oxidase, xanthine oxidase, the mitochondrial electron transport chain, and uncoupled endothelial nitric oxide synthase [13]. A study by Sigala et al. found higher eNOS phosphorylation and expression in human stable plaques compared with unstable plaques. They argue that preserved eNOS signaling is associated with plaque stability in humans [14]. Nitric oxide (NO) is an endothelial vasodilator that also prevents lipoprotein oxidation, smooth muscle cell growth, and inflammation. As a result, reduced NO bioavailability is a key component of endothelial dysfunction in the pathway to atherosclerosis [14]. Prominent risk factors, such as hypercholesterolemia, hypertension, diabetes mellitus, and smoking, are known to decrease endothelial nitric oxide (NO) and exacerbate ROS generation [13]. Oxidative stress and inflammation feedback loops have also been observed in tumor microenvironments (TMEs). A prospective longitudinal study on human colorectal cancer (CRC) patients found significantly elevated inflammatory markers and oxidative stress markers in patients compared with healthy controls. Catalase and reduced glutathione (GSH) were lower in CRC patients (*p* < 0.001), while oxidized glutathione (GSSH) and the GSSG/GSH ratio were higher (*p* < 0.001). CEA (*p* < 0.001) and CA 19.9 (*p* < 0.05) were higher in CRC patients. CRP was the only inflammatory marker not to show a significant difference. Treatment was associated with recovery of antioxidant enzyme activities and improvement in inflammation. The authors argue that this supports a relationship between these processes and human cancer [15]. Aberrant redox pathology is a potential upstream driver of both atherosclerotic and malignant processes. Oxidative stress may translate exposures such as smoking, metabolic dysfunction, and aging into aberrant inflammation, representing a convergence point of shared oxidative and inflammatory signaling in ASCVD and cancer [15].

Accumulating LDL in the subendothelial space is oxidized by ROS, rendering it highly atherogenic. Endothelial cells internalize the oxidized LDL via lectin-like oxidized LDL receptor-1 (LOX-1), intercellular adhesion molecule-1 (ICAM-1) and vascular cell adhesion molecule-1 (VCAM-1) [16]. Monocyte chemoattractant protein-1 (MCP-1) recruits monocytes to the endothelial lining. This oxidative stress and LOX-1 activation lead to amplification of inflammatory pathways involving nuclear factor kappa B (NF-κB) and other factors, which upregulate proinflammatory cytokines, chemokines, adhesion molecules, and matrix metalloproteinases [17,18]. Viewing atherosclerosis through a chronic inflammatory lens may uncover a potential overlap with malignancy.

Targeted anti-inflammatory therapy has shown promise in reducing cardiovascular events. The CANTOS trial observed that canakinumab, a monoclonal antibody against IL-1β, may reduce the primary composite endpoint by 15% [19]. The primary endpoint was a first occurrence of any one of three events: nonfatal MI, nonfatal stroke, or cardiovascular death. Their secondary endpoint, which added hospitalization for unstable angina requiring revascularization, was 17% lower compared to the control group. This landmark trial had a secondary observation that the magnitude of benefit was associated with the degree of inflammation reduction [19]. The LoDoCo2 trial found that colchicine may reduce cardiovascular events by 31% for patients with stable atherosclerosis, while the COLCOT trial found a reduction of 23% for patients after recent myocardial infarction (MI) [20]. These landmark trials help support a potential causal relationship between anti-inflammatory therapy and reduced ASCVD.

Though intended to investigate the cardiovascular benefits of IL-1β inhibition, the CANTOS trial secondarily found a 67% reduction in lung cancer incidence associated with 300 mg canakinumab treatment [21]. The authors propose that IL-1 may facilitate cancer invasion by inducing matrix metalloproteinase (MMP)-2, which allows cancers to cross the basement membrane. Inhibition of IL-1 by canakinumab may prevent metastatic spread, while also reducing atherosclerotic risk. This is especially notable when considering that commonly used cancer therapies have a negative effect on cardiovascular health [22]. While the evidence lays groundwork for further investigation, the primary endpoints of COLCOT and LoDoCo2 were exclusively cardiovascular. Cancer incidence was not a prespecified endpoint in either trial, making this an exploratory or observational finding.

## 4. Inflammation in Cancer Development and Progression

From the beginning of carcinogenesis to metastasis, inflammation may play an important role [23]. Though acute inflammation may trigger an anticancer immune response, chronic inflammation may contribute to immunosuppression, creating an environment favorable to carcinogenesis [24]. A review by Akkız H. et al. synthesized the literature on signaling pathways (NF-κB, JAK-STAT, MAPK, PI3K/AKT) and inflammatory mediators (cytokines, chemokines) that may link inflammation and carcinogenesis [24]. An editorial by Mishra S. et al. described that research has focused on the initiation mechanisms, while understanding of inflammation-driven metastasis remains limited [25]. Primary tumor cells invade locally, enter the circulation, lodge in microvessels in distant tissues, invade parenchyma, and form micrometastatic deposits, some of which develop into macroscopic metastases [26]. Classic examples of inflammation-associated cancers include HPV-associated cervical cancer, H. pylori-driven gastric cancer, hepatitis-related hepatocellular carcinoma, IBD-associated colorectal cancer, and asbestos-induced mesothelioma [27,28,29,30,31].

A meta-analysis of 107 epidemiological studies suggested that the common thread linking these diverse cancers may be the presence of inflammatory mediators in their respective TMEs [32]. Of the 19 meta-analyses performed, 10 showed statistically significant positive associations between markers and cancer incidence. The strongest association was between lung cancer and CRP (hazard ratio of 2.03). Notably, CRP was the most consistently associated marker, including with CRC, despite the study by Acevedo-León D. et al. finding CRP to be the only marker to not show a significant difference [15]. The strength of the evidence in favor of CRP’s association, however, is more robust, as demonstrated by larger sample sizes [32]. Chemokines and cytokines play complex roles in cancer due to their ability to both suppress and promote tumor progression. A review by Abdul-Rahman T et al. argued that while pro-inflammatory cytokines like IL-6 and TNF-α potentially facilitate tumor proliferation, immunostimulatory cytokines such as IL-12, IL-2, and IFN-α may enhance cancer-cell identification by cytotoxic effector cells and recruit antitumor immune responses [33]. Several of these immunostimulatory cytokines, including GM-CSF, IL-7, IL-12, IL-15, IL-18, IFN-α and IL-21, have entered clinical trials for advanced cancer, and the FDA has approved IFN-α and IL-2 [34]. Nonetheless, low efficacy and dose-limiting toxicity limit these agents’ full potential. Two systematic reviews explored how immunostimulatory cytokines associate with tumor progression. Fu et al. found that while cytokines may have antitumor mediating properties, their clinical translation remains limited by pleiotropic biological effects, short half-lives, and dose-limiting toxicities. Refined treatment regimens and engineering strategies may help widen the therapeutic windows. Pasquali et al. pooled data from seven randomized controlled trials (RCTs) and found that biochemotherapy (chemotherapy + IFN-α + IL-2) was associated with higher progression-free survival than chemotherapy alone in metastatic melanoma. However, they found no significant improvement in overall survival. Furthermore, they identified higher toxicity rates with biochemotherapy [35]. Chemokine-cytokine combinations show promise by enhancing lymphocyte activation and co-stimulatory molecules, opening the door to potential anticancer combination therapies that may reverse immune evasion and redirect immunosuppressive cells [34,35].

Central to the pro-tumorigenic inflammatory pathways is NF-κB, a key regulator of inflammation and immune responses [36]. NF-κB operates in both directions, promoting cancer development and driving ASCVD progression. It may also orchestrate apoptosis resistance, metastasis, angiogenesis, and therapy resistance in cancer cells. Both canonical and noncanonical NF-κB pathways mediate apoptosis resistance in lymphomas by upregulating anti-apoptotic targets such as Bcl-xL, FLIP, cIAP, and XIAP. Inhibition of either pathway may restore sensitivity to apoptosis inducers and reduce expression of these survival factors [37]. An in vitro study on human glioblastoma cell lines found a direct correlation between NF-κB activation and angiogenesis in glioblastoma [38]. NF-κB promotes angiogenesis by inducing expression of VEGF, IL-8, and other factors that stimulate endothelial cell proliferation and blood vessel formation essential for tumor growth and metastasis [38]. It may contribute to therapeutic resistance by upregulating multidrug resistance proteins (MDR1), DNA repair enzymes, and anti-apoptotic factors, while simultaneously modulating the TME to evade immune surveillance and treatment-induced cell death [39]. This data, however, remains largely preclinical and requires more extensive study before clinical applicability can be determined for NF-κB targeted therapy.

## 5. Mechanistic Links Between ASCVD and Cancer

Among validated shared inflammatory biomarkers is high-sensitivity C-reactive protein (hsCRP), whose circulating levels may help assess systemic inflammatory burden. Levels above 3 mg/L tend to suggest elevated inflammatory cardiovascular risk. A statement by The American College of Cardiology (ACC) recommends that a single measurement above this threshold can be used to identify asymptomatic patients with inflammatory risk [40]. Evidence is strong for hsCRP as a predictor of cardiovascular risk with prognostic value comparable to traditional risk factors. The 3 mg/L threshold is a viable clinical simplification endorsed by the ACC, though the risk relationship is continuous. Furthermore, the ACC guidelines also state that persistently elevated hsCRP levels should be monitored for therapy elevation [40]. A single measurement may be used for initial risk screening in non-acutely ill patients, with confirmation recommended before therapeutic decisions.

Beyond its cardiovascular risk stratification, hsCRP is an independent predictor of cancer incidence and mortality. In patients with CVD, a cohort study identified low-grade systemic inflammation measured by CRP levels up to 10 mg/L as a risk factor for incident cancer, most markedly lung cancer [41]. Hepatic CRP is upregulated by IL-6, which is similarly established for CVD and cancer prognosis assessment [40,42]. An inflammation reduction trial found that the highest quartile of IL-6 elevation was associated with a 2.11-fold increase in CVD event risk [43].

The IL-1 inflammatory family includes tumorigenicity 2 (ST2), which may reflect heart failure (HF) risk and a variety of cancers. Elevated ST2 is associated with HF both in the acute and chronic settings. Cardiac remodeling and disease severity may also be reflected by ST2 elevations. Its effects on immune cell recruitment have the potential to impact tumor progression. A cross-sectional case–control biomarker study found serum ST2 levels to be significantly elevated in breast cancer patients. They also found that in ER-positive breast cancer specifically, ST2 levels are significantly associated with factors that indicate poor prognosis [44]. Another biomarker study revealed that elevated ST2 was independently associated with poorer survival in patients with advanced pancreatic ductal adenocarcinoma [45]. A meta-analysis analyzing case–control studies found that serum ST2 and ST2 mRNA levels were significantly elevated in hepatocellular carcinoma patients [46]. This was the largest supporting study; however, evidence remains moderately strong. Large prospective validation studies and randomized trials are needed to confirm prognostic utility.

Clonal hematopoiesis of indeterminate potential (CHIP) occurs when somatic mutations develop in leukemia-associated driver genes (DNMT3A, TET2, ASXL1, JAK2), although in the absence of hematologic malignancy [7]. CHIP is known to correlate with increased all-cause mortality and risk of coronary heart disease and ischemic stroke [47].

ASCVD and cancer appear to converge on a network of chronic inflammation, endothelial dysfunction, oxidative stress, immune dysregulation, and tissue remodeling. Shared upstream exposures such as aging, smoking, and obesity may exacerbate these interconnected processes. The anti-tumor p53 regulates the cell cycle, and its disruption is implicated in both cancer and atherosclerosis. Wild-type p53 can accumulate in atherosclerotic plaques and co-localize with inflammation and smooth muscle cell proliferation. Deficiency of p53 in knockout mice was associated with exacerbated atherosclerosis and more than tripled local cell proliferation [48,49]. Other cell growth regulatory genes, such as pRb, p15, p16, cyclins, CDK2, and CDK4, are associated with plaque-mediated stenosis and post-angioplasty restenosis. This trend is similar to their roles in cancer cell cycle dysregulation [50,51,52]. CHIP is fundamentally a shared upstream driver that independently promotes both ASCVD and malignancy. The overarching concept of ASCVD-cancer convergence has strong clinical evidence for shared inflammatory and genetic components, studied through the CANTOS trial and CHIP cohort studies. However, the specific role of p53 in atherosclerosis and the detailed pathways of bidirectional drivers have largely preclinical evidence.

## 6. Bidirectional Relationship: ASCVD Promoting Cancer

**Figure 1:**
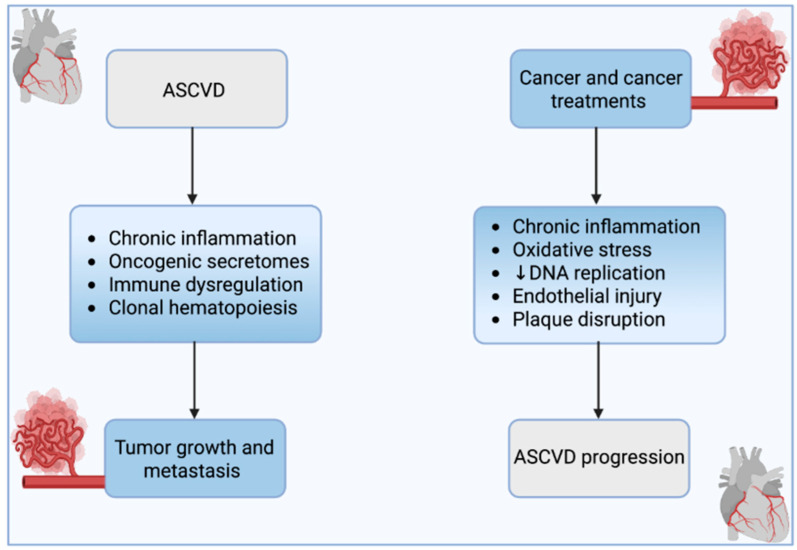
Summary of the bidirectional relationship between ASCVD and Cancer. Created in BioRender. Chughtai, S. (2026) https://BioRender.com/z011cpy, accessed on 29 June 2026.

Population-based cohort studies have found increasing evidence for an association between cardiovascular disease and subsequent cancer risk. An analysis of over 27 million patients demonstrated a 13% higher likelihood of cancer development in patients with CVD compared with healthy controls. This trend was especially prominent for atherosclerotic CVD patients. This suggests that the mechanisms specifically involved with atherosclerosis may contribute to oncogenesis (Figure 1) [53]. Reverse cardio-oncology studies have shown that CVD may promote pro-tumorigenic factor secretion (secretomes), neurohormonal activation, systemic inflammation, immune reprogramming, and oxidative stress [6].

### 6.1. Oncogenic Secretomes

Pathologies such as MI or hypertrophy may change the composition and concentration of cardiac-based bioactive molecules [54]. Serine protease inhibitor A3 (SerpinA3) is one such molecule found to be elevated in mouse models of infarction-induced HF compared to healthy controls. Furthermore, the severity of left ventricular dysfunction and scarring strongly coincided with tumor growth [42]. SerpinA3 activates the Akt-S6 phosphorylation pathway in human colon cancer cells, promoting proliferation. A knockout study of SerpinA3 in melanoma cells found that while it did not affect proliferation, it appeared to significantly lower cell migration and matrix invasion [55]. In glioblastoma brain tumor-initiating cells, SerpinA3 knockout was associated with lowered cell migration and invasion, as well as proliferation. This was found to increase survival in murine models [56,57]. Interestingly, SerpinA3 elevation appeared to reduce tumor progression in lung cancer, suggesting potential tissue-specific functions [58]. The evidence for SerpinA3 as a mechanistic link between HF and cancer promotion is predominantly preclinical, with limited and indirect human data. No clinical study has thus far prospectively measured SerpinA3 in HF patients and correlated it with subsequent cancer incidence or cancer outcomes.

The post-MI period shows increased secretion of small extracellular vesicles (sEVs), which contain tumor-promoting cardiac mesenchymal stromal cells (cMSCs). Mouse models showed that cMSC-sEVs were most elevated in post-MI lung and colon cancer cell lines, and contained elevated levels of microRNAs [59]. Among these microRNAs, miR-22-3p is transferred through exosomes into tumor cells, where it may then inhibit iron-dependent lipid peroxidation, which is involved in ferroptosis-regulated cell death [60]. This has been observed in mouse models of xenograft tumors where MI was associated with decreased sensitivity to the ferroptosis activators erastin and imidazole ketone erastin [60]. More specifically, miR-22-3p targets a ferroptotic protein acyl-CoA synthetase long-chain family member 4 (ACSL4). When cardiomyocyte-specific miR-22-3p was inhibited, researchers found that tumor cells became more sensitive to ferroptosis, highlighting a potentially causal relationship [60]. The miR-22-3p/ACSL4/ferroptosis axis has been observed for lung cancer, osteosarcoma and diffuse large B-cell lymphoma [60,61]. Evidence is again largely preclinical. Human studies have not yet measured cMSC-sEVs in HF patients or correlated them with cancer outcomes. Yuan et al. observed that miR-22-3p is upregulated in plasma exosomes of HF patients; however, this was a biomarker observation and not a demonstration of functional tumor promotion in humans [60]. Medications typically used for cardiovascular management have shown underappreciated anticancer benefits. Spironolactone was found to significantly reduce cMSC-sEV levels, leading to suppressed post-MI tumor growth [59]. Clinical observational evidence for spironolactone’s association with reduced cancer risk is substantial. A systematic review and meta-analysis found that spironolactone use was associated with no increased cancer risk, while also correlating with a reduced risk of prostate cancer [62].

Another cardiac secretome that has been shown to function in oncogenesis is periostin. Its cardiac and serum levels become elevated in states of cardiac hypertrophy and remodeling, which has been associated with increased breast cancer cell growth [63]. By binding to cancer cells at integrin receptors, periostin may initiate oncogenic signaling cascades through the PI3K/Akt and FAK pathways [64,65]. Periostin elevation has been associated with multiple different malignancy types, including cancer of the breast, lung, ovary, and glioblastoma [64]. Human evidence for periostin in cardiac hypertrophy and remodeling is strong, while its role in cancer progression requires more extensive research to establish.

Distant tumors may utilize the tumorigenic environment created by cardiac secretomes, as activating transcription factor 3 (ATF3) overexpression triggers their secretion into systemic circulation [54]. Transgenic mouse models of cardiac hypertrophy with elevated ATF3 expression showed that tumors grew larger and more aggressively than tumors in control group mice. The researchers also found that both HF and cancer cell implantation were separately associated with elevated serum tumorigenic cytokine factor levels. They suggest that immune-mediated cross-talk between the heart and the tumor occurs, leading to progression of cancer and HF [54]. While the specific ATF3-mediated mechanism is preclinical, the overarching concept that HF promotes cancer is supported by multiple large human studies [7].

A wide variety of other cardiac-derived factors have been shown to potentially contribute to oncogenesis (Table 1). The vast diversity of these factors implies that targeting an individual component may be insufficient for managing the oncogenic effects of CVD [54].

### 6.2. Immune Dysregulation

**Figure 2:**
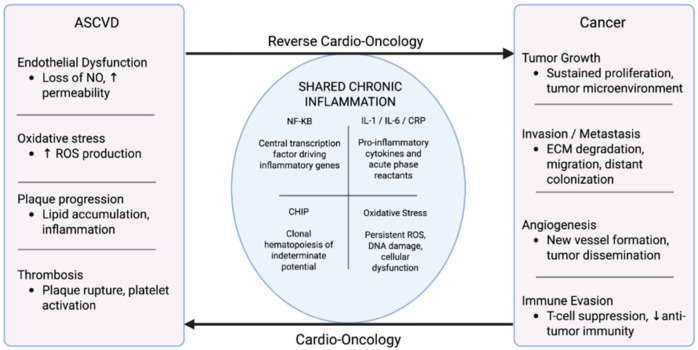
Comprehensive model of the bidirectional mechanisms connecting ASCVD and cancer. Created in BioRender. Chughtai, S. (2026) https://BioRender.com/e095s2u, accessed on 29 June 2026.

Immune cell reprogramming in CVD may lead to immunosuppression and decreased tumor immunosurveillance [63]. This has been seen in mouse models where post-MI cMSC-sEVs transformed macrophages into a tumor-promoting state. Researchers found that these macrophages upregulated programmed death-ligand 1 (PD-L1), which may facilitate apoptosis of cytotoxic T lymphocytes, allowing for tumor cell evasion of the immune system [59] (Figure 2).

## 7. Bidirectional Relationship: Cancer and Its Treatments Promoting ASCVD

Chemotherapy, immune checkpoint inhibitors (ICIs), and radiation therapy have all been shown to potentially exacerbate atherosclerosis through endothelial dysfunction, oxidative stress, inflammation, and plaque disruption (Figure 1).

### 7.1. Radiation and Chemotherapy

Radiation-associated ASCVD operates through endothelial and vascular injury. Free radicals are generated by ionizing radiation, leading to a proinflammatory state. In a process to replace damaged coronary intima, platelet aggregation, thrombosis, and plaque rupture are triggered [67]. Studies have found a dose-dependent linear relationship between radiation and ASCVD, with no clear safe threshold dose [68,69].

Anthracyclines have an established role in cardiomyopathy and may contribute to the onset of atherosclerosis by causing endothelial dysfunction. In a retrospective cohort study on patients aged 21 or less, anthracyclines were associated with increased dyslipidemia [70]. A retrospective analysis on 1934 breast cancer patients found similar increases in atherosclerosis risk with anthracycline treatment [71]. Long-term data on atherosclerotic risk specifically attributable to anthracycline-induced dyslipidemia remain limited. In a multicenter cohort study (*n* = 2356, median follow-up 14.2 years), diffuse large B-cell lymphoma patients treated with anthracycline-based chemotherapy experienced increased risk of HF; however, they found that coronary artery disease risk was actually decreased compared to the general population [72]. This suggests that the dominant clinical cardiovascular manifestation of anthracycline treatment may be cardiomyopathy or HF rather than clinical atherosclerotic events.

A variety of mechanisms have been proposed for anthracycline-induced cardiac side effects. Anthracyclines work by inhibiting topoisomerase II, which leads to fatal double-strand DNA breaks in cancer cells. There are two common subtypes of topoisomerase II in human cells: topoisomerase IIα (TOP2α) in rapidly proliferating cells, including cancer cells, and topoisomerase IIβ (TOP2β) in quiescent cells, including cardiomyocytes. The anthracycline effect on TOP2α is the primary pathway for its antitumor function, while the effect on TOP2β is the pathway for cardiotoxicity [73,74]. TOP2β disruption leads to DNA double-strand breaks in the quiescent cardiomyocytes. This activates the p53 response and results in cardiomyocyte apoptosis. Studies have also shown that Doxorubicin-bound TOP2β may inhibit the expression of other genes, including peroxisome proliferator-activated receptor-γ coactivators (PGC-1α and PGC-1β), which are important for mitochondrial function. This leads to mitochondriopathy and ROS generation classically associated with anthracycline cardiotoxicity [75]. Further corroborating this, cardiomyocyte deletion of TOP2β in mice was associated with increased resistance to HF in the setting of doxorubicin treatment [74].

5-Fluorouracil (5-FU) is typically used for management of gastrointestinal and breast cancers. It inhibits thymidylate synthase, which leads to insufficient thymidine for DNA replication. As an analog of uracil, 5-FU also has cytotoxic effects when incorporated into RNA and DNA [76,77]. 5-FU has been associated with risk of coronary vasospasm; however, its contribution to coronary atherosclerosis requires further study. Mechanistically, 5-FU may upregulate the activity of endothelin-1, which increases vascular smooth muscle contractility leading to vasospasm. Direct damage to endothelial cells has also been suggested [78,79,80]. As per the current evidence, 5-FU treatment should be monitored and optimized for acute vasospasm management rather than plaque development.

### 7.2. Immune Checkpoint Inhibitors

ICIs may accelerate atherosclerotic plaque progression through T-cell-driven vascular inflammation. The PD-1/PD-L1 and CTLA-4 pathways normally downregulate vascular inflammation. Without this control, plaques undergo increased destabilization [81]. Imaging studies have identified a preference for ICIs appearing to target less-calcified plaques. A case–control imaging study of 40 ICI-treated lung cancer patients found that the annual progression rate for noncalcified plaque volume was 7 times higher in the ICI group [82].

Two classes of tyrosine kinase inhibitors (TKIs) act through distinct mechanisms to potentially promote ASCVD: ABL-directed TKIs (nilotinib, ponatinib) directly accelerate atherosclerosis, while the vascular endothelial growth factor (VEGF) directed TKIs (sunitinib, sorafenib, lenvatinib) exacerbate hypertension and endothelial dysfunction [83]. This was observed in a retrospective cohort study on 159 renal cell carcinoma patients, 116 of whom (73%) developed cardiovascular toxicity. Among sunitinib-treated patients, 65% developed some form of cardiovascular toxicity. This number dropped to 32% when excluding hypertension. Other VEGF inhibitors, including bevacizumab, sorafenib, and pazopanib, also showed high cardiovascular toxicity incidences ranging from 51% to 68% [83]. This study has strong systematic methodology; however, it is limited by its single-center design and modest sample size.

The c-Abl kinase is important for Tie2 receptor expression and angiopoietin-1-mediated endothelial cell survival. TKI-induced Abl kinase inhibition may contribute to endothelial damage and vascular dysfunction. Nilotinib and ponatinib have also been shown to increase platelet activation and thrombogenesis over collagen [84,85,86].

A large meta-analysis covering 72 RCTs found clinically relevant increases in MI, hypertension, proteinuria, and thromboembolism risk with VEGF inhibitors for cancer treatment. Notably, no significant increase in stroke was found [87]. A preclinical study showed that VEGF inhibition is associated with a 33% increase in atherosclerotic lesion load. Furthermore, mitochondrial function may be affected, as there was an increase in superoxide generation and uncoupling of eNOS [88]. Downstream inhibition of the PI3K-Akt pathway likely contributes to eNOS dysfunction and hypertension.

VEGF normally helps to maintain the capillary network. Inhibition of this can lead to a structural decrease in microcirculatory vessels, which subsequently increases vascular resistance and blood pressure. Endothelial cell integrity is also disrupted, leading to endothelial layer damage and uncovering of the matrix below. This contributes to thrombosis and blood loss [83]. Despite these adverse effects, VEGF inhibitor recipients showed lower overall mortality rates, demonstrating their anticancer efficacy and the need to weigh risks and benefits [87].

## 8. Therapeutic Implications

### 8.1. Anti-Inflammatory Strategies

Statin medications can inhibit NF-κB, reducing downstream inflammatory cytokines. They also appear to stabilize atherosclerotic plaques and support endothelial function, conferring them a dual therapeutic role for ASCVD and cancer [85,86]. Specifically with anthracycline use, a meta-analysis demonstrated that statin therapy reduced (*p* < 0.001) cardiotoxic side effects across seven RCTs [89]. They found a 54% relative risk reduction compared with placebo [89]. Inhibiting cholesterol-dependent pathways used by tumor cells to proliferate may be a primary mechanism [90]. Evidence for statin cancer benefits, however, is largely observational and varied. A target trial emulation using data from the ASPREE randomized trial found that statin cancer risk benefits varied by cancer type and statin lipophilicity. They found lipophilic statins to be associated with reduced cancer risk; however, hydrophilic statins did not show any association. Cancer of the prostate and breast showed significant associations; however, no significant associations were observed for colon, lung, and melanoma [90]. Primary endpoints of the ASPREE trial included disability-free survival, cardiovascular events, and all-cause mortality. Cancer was a prespecified secondary endpoint of ASPREE; however, the statin-cancer analysis by Debele et al. was a post hoc observational study.

The NLRP3 pathway, which involves the IL-1β–IL-6 cascade, is a well-documented inflammatory pathway shared by ASCVD and cancer [91]. IL-1β inhibition by canakinumab was associated with a significant decrease in CVD events and lung cancer risk. These, however, occurred at different doses, with 150 mg for CVD benefit and 300 mg for lung cancer benefit [92]. The lung cancer findings were based on exploratory analysis in a trial not powered for cancer outcomes, making the dose-specific cancer signal an unconfirmed association. Furthermore, the infection risk with canakinumab use suggests a need for careful monitoring and potential isolation. More research on which patient groups stand to benefit from canakinumab, along with careful dosing guidelines for each disease phenotype and infection mitigation, is required.

A novel IL-6 ligand inhibitor that may significantly reduce CRP and fibrinogen is ziltivekimab. This human monoclonal antibody was shown to reduce CRP by up to 92%, providing promising benefits for high-risk ASCVD patients [93]. It is particularly attractive because IL-6 occupies a central position within the inflammatory cascade linking ASCVD, clonal hematopoiesis, and cancer biology. Ziltivekimab’s anti-inflammatory potency is supported by the RESCUE trial, which enrolled CKD patients with hsCRP ≥2 mg/L. At 12 weeks, ziltivekimab was associated with a decrease in median hsCRP by 77% (7.5 mg), 88% (15 mg), and 92% (30 mg) compared to 4% with placebo (*p* < 0.0001). Dose-dependent reductions in other markers including fibrinogen, serum amyloid A, haptoglobin, secretory phospholipase A2, and lipoprotein were also found [93]. Secondary analysis has associated ziltivekimab with reduced neutrophil-lymphocyte ratio (NLR), which is an established marker of malignancy risk [94,95]. While RESCUE was a rigorous phase 2 RCT conducted in humans, its primary endpoint was percentage change in hsCRP at 12 weeks rather than cancer or cardiovascular outcomes. The mechanistic rationale is strong; however, there is currently no direct clinical evidence of cancer benefit from ziltivekimab treatment.

Another anti-inflammatory treatment modality of interest is colchicine. This medication also targets NLRP3 to reduce downstream IL-1β, IL-6, and CRP [96,97]. A meta-analysis involving 22,983 participants showed evidence of reduced MI and stroke with colchicine treatment [98]. They found that colchicine treatment was associated with reduced MI (RR 0.74; ARR of 9 fewer events per 1000 patients) and stroke (RR 0.67; ARR of 8 fewer events per 1000 patients) [98]. Long-term assessments on cancer risk showed no adverse effects, with some data suggesting reduced colorectal cancer and hepatocellular carcinoma risk [99,100]. Furthermore, a multicenter cohort study showed that colchicine may have potential in myelodysplastic syndrome and acute myeloid leukemia risk reduction. The researchers proposed anti-inflammatory modulation of clonal hematopoiesis as a potential mechanism [101]. The cardiovascular benefit of colchicine for MI and stroke has strong clinical evidence; however, benefit for cancer risk has indirect and observational evidence. No prospective RCT has tested colchicine with cancer incidence as a primary endpoint, making this an important next step based on the mechanistic plausibility. Furthermore, multiple cardiovascular trials have reported neutral effects on overall cancer incidence, indicating that anticancer benefit may be modest, cancer-specific, or limited to selected high-risk populations [102,103]. The absence of dedicated randomized oncology studies remains a barrier to recommending colchicine for dual cardiovascular-cancer prevention.

A central consideration in the use of anti-inflammatory strategies for both ASCVD and cancer is the preservation of immune function. In the CANTOS trial, canakinumab was associated with an increased rate of fatal infection [19]. In this context, colchicine, which primarily targets neutrophil function, and ziltivekimab, which focuses on IL-6 signaling, may have more limited immunosuppressive effects compared with broader IL-1β inhibition [40]. IL-6 signaling has context-dependent roles in host defense and tumor immunology, raising theoretical concerns that long-term suppression could impair immune surveillance in certain patient populations [93]. Consequently, enthusiasm for ziltivekimab should be balanced by recognition that its proposed role in cancer prevention remains speculative and unsupported by prospective oncology trials. Cancer incidence was not a prespecified endpoint of the CANTOS trial. Further data are needed to define optimal use and safety across different clinical contexts. The current evidence base remains uneven, with promising mechanistic hypotheses having yet to translate into consistent clinical benefit. Importantly, reductions in inflammatory biomarkers should not automatically be interpreted as reductions in cancer incidence or mortality.

### 8.2. CHIP as a Therapeutic Target

Somatic mutations in driver genes such as DNMT3A, TET2, ASXL1, and JAK2 may promote both accelerated atherosclerosis and premalignant hematopoietic growth. CHIP offers a critical convergence point between ASCVD and cancer [104].

According to the American Heart Association’s scientific statement on CHIP, the syndrome is linked to a 40–50% increase in all-cause mortality, with coronary heart disease and ischemic stroke accounting for most of this increase [104]. Mechanistically, CHIP-associated mutations, especially in TET2, increase IL-1β-mediated cytokine signaling and NLRP3 inflammasome activation, suggesting that inflammation may be a common risk factor for both cancer and cardiovascular disease [104]. Although such treatments are still under development, these findings offer a physiologic justification for targeting inflammasome or cytokine pathways in this population.

Individualized optimization of traditional risk factors using guideline-directed prevention strategies is currently at the center of management [104,105]. Optimizing traditional risk factors is extrapolated from general cardiovascular prevention guidelines, not from trials enrolling CHIP carriers. The clonal hematopoiesis risk score (CHRS), developed to stratify risk for myeloid neoplasms, has also been associated with cardiovascular mortality in older adults, highlighting its potential utility beyond oncologic risk assessment [105]. Emerging strategies such as epigenetic modulators and pathway-specific agents targeting CHIP driver mutations are being explored [104]. Given the shared mechanisms, integrated approaches to study design incorporating both cardiovascular and oncologic endpoints may be important in future trials.

Dedicated CHIP clinics support risk-based surveillance and management [104]. A 2026 AHA scientific statement supported a multidisciplinary approach, combining CHIP clinic evaluation and preventive cardiology for optimization of cardiovascular therapies and clonal stability [104]. These findings support a model of integrated care addressing both cardiovascular and oncologic risk.

Despite growing interest, the clinical utility of CHIP clinics remains incompletely established. There is a lack of consensus regarding optimal screening strategies and surveillance intervals. Importantly, identification of CHIP rarely changes management beyond aggressive implementation of established cardiovascular prevention measures [104]. Although observational data suggest that certain CHIP genotypes may preferentially activate inflammatory pathways such as NLRP3 and IL-1β signaling, no targeted therapies have yet demonstrated outcome benefits in randomized studies. Consequently, CHIP clinics should currently be used for risk stratification and counseling rather than verified clinical therapies [106].

### 8.3. Integrated Prevention Strategies

Fundamental strategies for lowering the risk of ASCVD and cancer include lifestyle changes that target common inflammatory pathways. Guidelines and results from randomized trials encourage anti-inflammatory eating habits [40]. Following a Mediterranean diet was linked to lower rates of CVD events and inflammatory biomarkers in the PREDIMED trial [107]. Sustained decreases in inflammatory markers were also shown by long-term follow-up [107]. Meta-analyses of randomized studies demonstrated consistent decreases in inflammatory biomarkers with adherence to the Mediterranean diet [108].

Mediterranean diets have been linked to lower cancer risk in addition to their cardiovascular benefits. Positive lifestyle habits may lower incidences of kidney, hepatocellular, colorectal, and obesity-related malignancies [109]. In keeping with this, recommendations for preventing cancer place a strong emphasis on plant-based eating habits, exercise, and controlling weight, all of which are linked to improved metabolic profiles, decreased oxidative stress, and lower levels of systemic inflammation. A large meta-analysis of 126 studies found statistically significant but modest risk reductions from Mediterranean diets for cancer. For example, RR for CRC and liver/gallbladder cancer was 0.95 and 0.94, respectively [110].

Another crucial anti-inflammatory strategy is physical activity. To lower chronic low-grade inflammation, the American College of Cardiology advises engaging in at least 150 min of moderate-intensity or 75 min of strenuous cardiovascular activity per week [40]. Frequent exercise reduces resting CRP in several ways, such as decreased adipose tissue cytokine production, greater production of specific pro-resolving mediators, and improved endothelial function [40]. While RCTs have demonstrated CRP reduction with exercise, the link between exercise-induced inflammation reduction and cardiovascular endpoints is largely derived from observational studies, making causal inference unreliable [111]. Since smoking is a significant cause of chronic inflammation, ceasing to smoke is crucial [40]. A longitudinal study found that CRP reduction was seen only after 4 years of follow-up since smoking cessation. The benefits may not be evident in the short term [112].

Aggressive management of traditional cardiovascular risk factors, including dyslipidemia, hypertension, diabetes, and obesity, remains essential, particularly among cancer patients and survivors [90]. Notably, cancer survivors are more likely to discontinue statin therapy despite clear indications, highlighting a gap in care. Integration of cardiovascular risk assessment into oncologic practice, and vice versa, represents an important step toward comprehensive management of shared inflammatory risk.

Although pharmacologic and lifestyle-based therapies show promise in the treatment of cardiovascular disease, their effect on cancer outcomes is less clear (Table 2). A strategy focused on known cardiovascular risk reduction, with consideration of broader inflammatory processes, is supported by the literature. To determine whether focused anti-inflammatory strategies offer reliable advantages in both diseases, further studies are required.

## 9. Limitations and Clinical Applicability

This narrative review utilizes evidence from varying levels of reliability, ranging from randomized trials to retrospective cohorts, registry studies, and preclinical experiments. Much of the available evidence for the link between ASCVD and cancer is observational, which supports association but limits definitive causal inference. Mechanistic data are largely animal models or translational study-based, decreasing applicability to human biology. ASCVD and cancer are heterogeneous conditions, for which one cardiovascular or tumor phenotype may not be generalizable. Several of the therapeutic signals discussed are currently at a hypothesis stage rather than clinically applicable. Temporal relationships are not always clear, complicating the assessment of whether one disease directly promotes the other or arises from shared processes. Sex, race, and ethnicity-specific variables are underrepresented in many cohorts, limiting the external validity of the findings. Finally, there is currently a dearth of long-term prospective trials with dual cardiovascular and oncologic endpoints.

The strongest human evidence for inflammation as a cause of ASCVD is supported by randomized trials including CANTOS and colchicine studies. Oncology outcomes, however, are exploratory rather than primary endpoints. Epidemiologic evidence for reverse cardio-oncology is also largely observational currently. Mechanistic evidence is strongest for IL-1β/IL-6 signaling, CHIP-associated inflammation, and endothelial injury from cancer therapies. Secretomes and individual biomarkers have weaker evidence as data are largely preclinical or utilize small cohorts. Canakinumab has contradictory evidence for lung cancer benefit, which was unable to be replicated in three subsequent oncology trials [115,116,117].

## 10. Conclusions and Future Work

A clinically significant bidirectional link between cancer and ASCVD has been established in the literature, with chronic inflammation as a common association. Inflammatory signaling facilitates endothelial dysfunction, oxidative stress, lipid retention, plaque progression, and thrombosis in ASCVD, while these same pathways may contribute to tumor initiation, immune evasion, angiogenesis, invasion, and metastasis. Mediators such as NF-κB, IL-1β, IL-6, CRP, oxidative stress pathways, and clonal hematopoiesis overlap, indicating that these illnesses may be linked to expressions of systemic inflammatory dysregulation. Clinical studies show that cancer survivors and patients receiving cardiotoxic medications exhibit accelerated vascular disease, while individuals with CVD appear to have increased cancer.

Multiple medications show potential for reducing risk across both cancer and ASCVD. These include statins, colchicine, canakinumab, and IL-6-targeted drugs. Biomarker-guided prevention, integrated cardio-oncology models, and studies with dual cardiovascular and oncologic objectives may support future advancements. Utilizing inflammation as a unifying process may help manage organ-specific diseases in two major causes of death worldwide.

Bidirectional associations have been shown in multiple epidemiological studies; however, further evidence of direct causality may help with treatment modulation. The CANTOS trial provided the strongest human evidence for a causal inflammatory link between these diseases; however, it was not designed as an oncology trial and cancer findings were exploratory [92].

Randomized controlled trials specifically for dual cardiovascular and oncologic endpoints would be more convincing. These studies should involve large-scale inflammatory biomarker categorization longitudinally to examine whether fluctuations in specific mediators hold clinical benefits for both diseases [118]. Validated biomarkers that consistently predict concurrent risk for ASCVD and cancer should be a focus. Mendelian randomization studies may provide additional support for causality by removing confounding lifestyle variables and focusing on shared genetic architecture. Infection risk observed with canakinumab highlights the importance of balancing anti-inflammatory benefits and immunosuppression risks [19]. Studies should compare the benefits and adverse effects of canakinumab with those of more narrow anti-inflammatory agents such as colchicine, which targets neutrophils, or ziltivekimab, which focuses on IL-6.

Future research should examine specific cancer type and CVD phenotype pairings. Instead of aggregate studies, these will help elucidate which precise inflammatory axes are relevant for each combination. Single-cell transcriptomic and spatial proteomic studies may help characterize these specific and granular processes at the interface of atherosclerotic plaques and TMEs.

Patient selection will also be important to characterize, as not all individuals with elevated inflammatory markers will benefit equally. The bidirectional relationship is likely modified by demographics including sex-specific biology, environment, and ethnic group. Sex differences arise in immunity and hormonal regulation of inflammation. Female patients exhibit larger inflammatory responses and biomarker levels than male patients [119]. Clinical application will require further understanding of when and in whom to intervene.

## Figures and Tables

**Table 1:** Oncogenic secretomes associated with ASCVD.

Secretome	Context in the literature	Mechanism of Action	Associated Cancer Types
SerpinA3 (Serine Protease Inhibitor A3) [42,55,56]	Elevated in infarction-induced HF. Severity correlates with LV dysfunction and scarring.	Activates the Akt-S6 phosphorylation pathway, promoting proliferation. Enhances cell migration and matrix invasion.	Colon cancer, melanoma, glioblastoma, lung cancer
Small Extracellular Vesicles (sEVs) containing cMSC-derived oncogenic cargo [59]	Increased secretion post-MI from cardiac mesenchymal stromal cells (cMSCs).	Deliver elevated levels of oncogenic cytokines, proteins, and microRNAs to tumor cells.	Lung cancer and colon cancers
miR-22-3p [60,61]	Transferred via exosomes from cardiomyocytes post-MI.	Inhibits ferroptosis by targeting ACSL4, blocking iron-dependent lipid peroxidation. Promotes cancer cell survival.	Lung cancer, osteosarcoma, diffuse large B-cell lymphoma
Periostin [63,64,65]	Elevated in cardiac hypertrophy and remodeling.	Binds integrin receptors on cancer cells, activating PI3K/Akt and FAK pathways. Promotes survival, proliferation, angiogenesis, metastasis, and chemoresistance.	Breast cancer, lung cancer, ovarian cancer, glioblastoma
ATF3-triggered secretomes [54,66]	ATF3 overexpression in cardiac hypertrophy triggers secretion into systemic circulation.	Immune-mediated cross-talk between heart and tumor. Elevates serum tumorigenic cytokine levels.	Breast cancer

**Table 2:** Treatment modalities showing promise for both ASCVD and cancer management.

Category	Therapy	Mechanism of Action	ASCVD Benefit	Cancer Benefit	Limitations
Anti-Inflammatory	Statins [113]	Inhibit NF-κB → reduce IL-1, IL-6, TNF-α, CRP	Plaque stabilization, endothelial protection	May reduce anthracycline cardiotoxicity; inhibit cholesterol-dependent tumor proliferation	Predominantly observational data for cancer benefit; lack of RCT evidence for cancer endpoints; varied data based on statin type and cancer type
Anti-Inflammatory	Canakinumab (IL-1β inhibitor) [91,92]	Inhibits IL-1β in the NLRP3/IL-1β–IL-6 cascade	May decrease CVD events (150 mg dose)	May reduce lung cancer risk (300 mg dose)	Increased risk of infection; lung cancer benefit has not been replicated in oncology trials
Anti-Inflammatory	Ziltivekimab (IL-6 ligand inhibitor) [93,94,95]	Monoclonal antibody inhibiting IL-6; reduces CRP, fibrinogen, and NLR	CRP reduction in high-risk ASCVD patients	May reduce colorectal cancer and hepatocellular carcinoma risk	No direct clinical evidence of cancer benefit
Anti-Inflammatory	Colchicine [96,98,99,100,101,114]	Targets NLRP3 inflammasome → reduces IL-1β, IL-6, CRP	May reduce MI and stroke risk	May reduce colorectal cancer, HCC, and MDS/AML risk	Lack of RCT data with cancer incidence as a primary endpoint
CHIP-Targeted	Guideline-directed risk factor optimization [104,105]	Individualized risk assessment; management of traditional CV risk factors	Primary and secondary prevention per guidelines	CHRS may stratify risk for myeloid neoplasm progression	Recommendation is based on general prevention guidelines, not from controlled trials on CHIP carriers
CHIP-Targeted	Epigenetic modulators, pathway-specific agents [104,106]	Target CHIP driver mutations (DNMT3A, TET2, ASXL1, JAK2) and downstream NLRP3/IL-1β signaling	May address CHIP-associated increase in all-cause mortality (CHD and ischemic stroke)	May mitigate premalignant hematopoietic clonal expansion	Largely preclinical or in early-phase trials
CHIP-Targeted	Multidisciplinary CHIP clinics [104,106]	Combined CHIP clinic evaluation and preventive cardiology	Greater optimization of cardiovascular therapies	Signals of clonal stability in cancer survivors with CHIP	Evidence base is observational and lacking outcomes data
Integrated Prevention	Mediterranean diet [40,109,110]	Anti-inflammatory dietary pattern; reduces hsCRP, IL-6, TNF-α	May lower rates of major CV events	May lower risk of kidney, hepatocellular, colorectal, and obesity-related cancers	Observational data and modest effect sizes
Integrated Prevention	Physical activity (≥150 min moderate or ≥75 min vigorous/week) [40,111]	Reduces adipose cytokines; increases pro-resolving mediators; improves endothelium	May lower resting CRP; ACC-recommended for CV risk reduction	May reduce cancer risk across multiple types	Largely derived from observational studies; lacking data on hard endpoints
Integrated Prevention	Smoking cessation [40,112]	Reduced proinflammatory cytokines; restored inflammation resolution	May reduce CV risk	May reduce cancer risk across multiple types	CRP reduction was not observed until after 4 years follow-up
Integrated Prevention	Aggressive traditional risk factor management (dyslipidemia, HTN, DM, obesity) [90]	Guideline-directed management of modifiable risk factors	Essential for CV risk reduction, especially in cancer patients/survivors	May reduce cancer risk across multiple types	Evidence primarily from longitudinal registries, not RCTs; heterogeneity across cancer types and treatments

## Data Availability

Not applicable.

## Abbreviations

The following abbreviations are used in this manuscript:

MDPI	Multidisciplinary Digital Publishing Institute
DOAJ	Directory of open access journals
TLA	Three-letter acronym
LD	Linear dichroism
